# A Bipartite Network-based Method for Prediction of Long Non-coding RNA–protein Interactions

**DOI:** 10.1016/j.gpb.2016.01.004

**Published:** 2016-02-22

**Authors:** Mengqu Ge, Ao Li, Minghui Wang

**Affiliations:** 1 School of Information Science and Technology, University of Science and Technology of China, Hefei 230027, China; 2 Centers for Biomedical Engineering, University of Science and Technology of China, Hefei 230027, China

**Keywords:** lncRNA, Protein, Interaction, Bipartite network, Propagation

## Abstract

As one large class of non-coding RNAs (ncRNAs), long ncRNAs (**lncRNAs**) have gained considerable attention in recent years. Mutations and dysfunction of **lncRNAs** have been implicated in human disorders. Many **lncRNAs** exert their effects through **interactions** with the corresponding RNA-binding **proteins**. Several computational approaches have been developed, but only few are able to perform the prediction of these **interactions** from a network-based point of view. Here, we introduce a computational method named lncRNA–protein **bipartite network** inference (LPBNI). LPBNI aims to identify potential lncRNA–interacting **proteins**, by making full use of the known lncRNA–protein **interactions**. Leave-one-out cross validation (LOOCV) test shows that LPBNI significantly outperforms other network-based methods, including random walk (RWR) and protein-based collaborative filtering (ProCF). Furthermore, a case study was performed to demonstrate the performance of LPBNI using real data in predicting potential lncRNA–interacting proteins.

## Introduction

An increasing number of studies show that approximately 2% of the whole mammalian genome represents protein-coding genes, whereas the majority of the genome consists of non-coding RNA (ncRNA) genes. ncRNAs had long been regarded as transcriptional noise, but recent investigations demonstrate that ncRNAs play an important role in the regulation of diverse biological processes [1], [2], [3], [4], [5]. Long ncRNAs (lncRNAs), which consist of more than 200 nucleotides, constitute a large class of ncRNAs [6], [7]. In the past several years, the number of identified lncRNAs has been increasing sharply because of the development of both bioinformatics tools and experimental techniques. Functional studies of lncRNAs show that mutated and dysfunctional lncRNAs are implicated in a range of cellular processes [8], [9], [10], [11], [12] and human diseases, ranging from neurodegeneration to cancer [13], [14], [15], [16], [17], [18]. Although some lncRNAs, *e.g.*, Xist [19] and MALAT1 [20], have been well studied, the functions of most lncRNAs remain unclear. Usually lncRNAs function through interacting with RNA-binding proteins (RBPs) [21], [22], [23], [24]. Therefore, it is important to predict the potential lncRNA–protein interactions, in order to study the complex function of lncRNAs.

Since the experimental identification of lncRNA–protein interactions remains costly, developing effective predictive approaches becomes essential. Recently, several computational methods have been reported for predicting potential lncRNA–protein interactions. For instance, Bellucci et al. developed catRAPID in 2011 [25] by taking into account secondary structure, hydrogen bonds, and van der Waals forces between lncRNAs and proteins. Next, Muppirala et al. [26] introduced a method named RPISeq, using only sequence information of lncRNAs and proteins. Support vector machine (SVM) classifiers [27] and random forest (RF) [28] are used to predict RBPs. In 2013, Lu et al. [29] developed a novel approach, named lncPro, which uses secondary structure, hydrogen bond, van der Waals force features, and yields the prediction score using Fisher’s linear discriminate method. Later on, an approach named RPI-Pred was developed by Suresh et al. [30], they trained SVM-based approach, by extracting sequence and high-order 3D structure features of lncRNAs and proteins.

All the aforementioned methods are based on the biological characteristics of ncRNAs and proteins. CatRAPID and lncPro combined sequence and structural features of lncRNAs and proteins. RPISeq was based on sequence features. RPI-Pred used the high-order structure features of lncRNAs and proteins. However some studies show that lncRNAs generally exhibit low sequence conservation [1], which may make it difficult to predict interactions based on the intrinsic properties of lncRNAs. Biological network-based methods are widely used in many types of studies, such as disease gene prioritization [31] and drug-target interaction prediction [32]. The development of bioinformatics technologies such as CLIP-seq and cross-linking immunoprecipitation, has enabled us to construct lncRNA–protein interaction networks. We introduce here a novel computational method, lncRNA–protein bipartite network inference (LPBNI), for the prediction of lncRNA–protein interactions. LPBNI identifies novel lncRNA–protein pairs by efficiently using the lncRNA–protein bipartite network. In order to evaluate the performance of the proposed method, we compared LPBNI with other network-based methods, including random walk (RWR) [31] and protein-based collaborative filtering (ProCF) [33]. RWR [31] has been used to predict genes associated with potential diseases. ProCF is derived from the recommendation algorithms, similar to the item-based collaborative filtering method [33]. The performance evaluation is based on leave-one-out cross validation (LOOCV) of the known lncRNA–protein interactions extracted from NPInter [34]. To further demonstrate the effectiveness of lncRNA–protein bipartite network, six lncRNAs were used to evaluate the performance of LPBNI in comparison with the existing methods, lncPro [29] and RPISeq [26]. These evaluation tests demonstrated that LPBNI outperforms the other methods significantly. In a case study, several potential interactions between lncRNAs and proteins identified by LPBNI were well supported by starBase [35], indicating the superior predictive ability of LPBNI.

## Results

### Performance comparison with other network-based methods on lncRNA–protein interactions prediction

We compared the performance of LPBNI with RWR [31] and ProCF [33]. ProCF is based on the idea that if a protein interacts with an lncRNA, similar proteins will be recommended as interacting with this lncRNA. The linkage between *p_i_* and *l_j_* can be defined as: ${\mathrm{score}}_{\mathrm{ij}}=\frac{\sum_{k=1\text{,}k\neq i}^{m}S_{P}(p_{i}\text{,}p_{k})a_{\mathrm{kj}}}{\sum_{k-1\text{,}k\neq i}^{m}S_{P}(p_{i}\text{,}p_{k})}$, where $S_{P}(p_{i}\text{,}p_{k})$ is the similarity between proteins *p_i_* and *p_k_*. Here, we used cosine vector similarity to measure the similarity of proteins: $S_{P}(p_{i}\text{,}p_{k})=\frac{\left|d(i)\cap d(k)\right|}{\sqrt{\left|d(i)||d(k)\right|}}$, where *d*(*i*) and *d*(*k*) are the degrees of proteins *i* and *k*, respectively.

**Figure 1:**
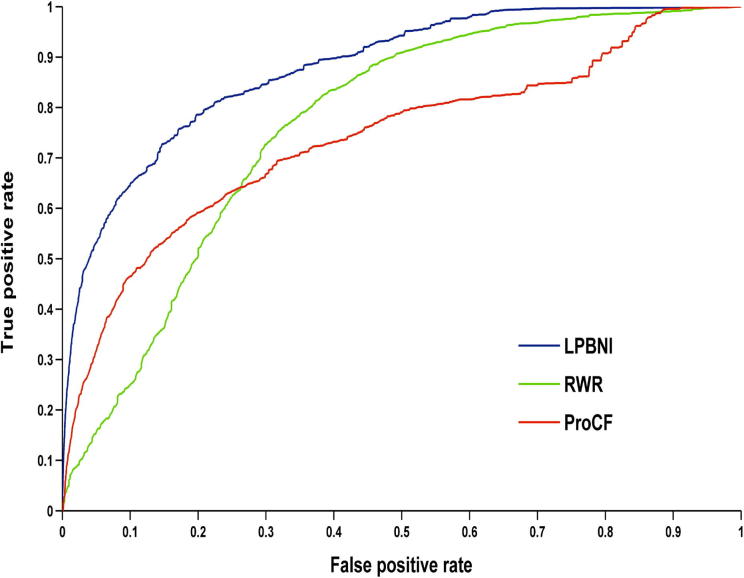
**Performance comparison of different methods using ROC curves in predicting lncRNA–protein interactions** Shown in the plot is the ROC for the whole dataset using LPBNI (blue, AUC: 0.878), RWR (green, AUC: 0.765), and ProCF (red, AUC: 0.738), respectively. LOOCV is implemented and 4796 known lncRNA–protein interactions are used as gold standard dataset. ROC, receiver operating characteristic; LPBNI, lncRNA–protein bipartite network inference; RWR, random walk; ProCF, protein-based collaborative filtering; AUC, area under this curve. LOOCV, leave-one-out cross validation.

We extracted 4870 lncRNA–protein interactions from NPInter 2.0 [34] (see “*Data collection and preprocessing*” for detail). In LPBNI, for one node, at least two interactions are required to perform LOOCV. Therefore, the nodes that have only one link are not considered in the performance evaluation, so we further get 4796 lncRNA–protein interactions which match that condition, and this dataset is taken as ‘gold standard’ data in the LOOCV test. The receiver operating characteristic (ROC) curves and the area under the curve (AUC) obtained using these methods are shown in Figure 1. It is obvious that LPBNI shows the highest true positive rate (TPR) at each false positive rate (FPR). In addition, the AUC value of LPBNI is 0.878 (Table 1), which is higher than that obtained using RWR (0.765) and ProCF (0.738), respectively. These data suggest that LPBNI has a better predictive ability compared with RWR and ProCF. To validate the reliability of LPBNI, we compared the sensitivity, accuracy, precision, and Matthew’s correlation coefficient (MCC) of LPBNI, RWR, and ProCF with specificities of 99.0% and 95.0%, respectively. As shown in Table 1, with specificity of 99.0%, sensitivity, accuracy, precision, and MCC of LPBNI are all higher than that with RWR and ProCF. When specificity was reduced to 95.0%, sensitivity and MCC increased for all three methods, with decreased precision, although the accuracy remained comparable. However, LPBNI still showed a higher performance in terms of sensitivity, accuracy, precision, and MCC, compared to RWR and ProCF.

The fold enrichment is also used to evaluate the performance of the proposed method, which can be defined as: *N*/2/*n* [37], where *N* represents the number of candidate proteins, and *n* is the ranking of the tested protein among the candidate proteins for the evaluation. Based on the formula, the average fold enrichments are 4.007, 3.590, and 1.653 for LPBNI, RWR, and ProCF, respectively. These data suggest that LPBNI outperforms the other methods in identifying lncRNA–related proteins with a higher rank. Table 2 shows the number of lncRNA–protein interactions that were correctly retrieved at 5%, 10%, 15%, 20%, and 50% of all the prediction results, respectively. Among 4796 true interactions between lncRNAs and proteins, LPBNI achieves a higher retrieval compared with RWR and ProCF, at each of the investigated percentiles. The biggest difference was observed for 5%, where LPBNI recovered 579 interactions successfully, and only 410 and 116 interactions were retrieved using RWR and ProCF, respectively.

**Supplementary Figure S1:**
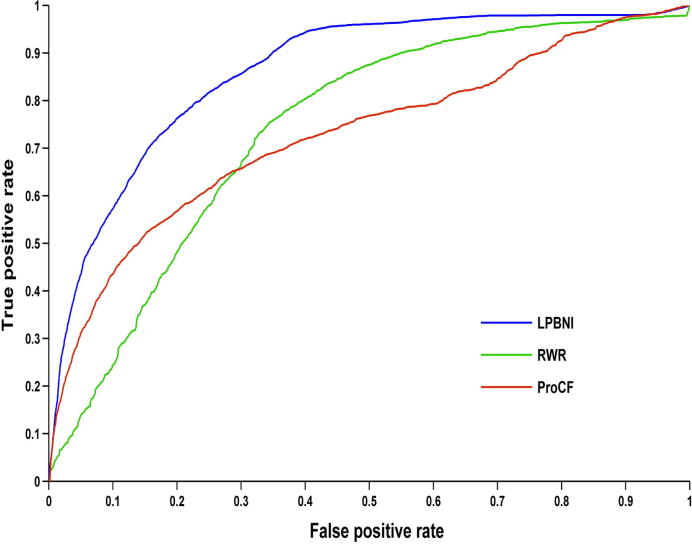
**Performance comparison of different methods using a 10-fold cross validation** Shown in the plot is the AUC value using LPBNI (blue), RWR (green), and ProCF (red), respectively. 10-fold cross validation is implemented and 4796 known lncRNA−protein interactions are used as the gold standard dataset.

Furthermore, 10-fold cross validation was applied, in order to conduct a comprehensive performance evaluation of LPBNI in predicting lncRNA–protein interactions. All lncRNA–protein interactions (4796) are randomly divided into 10 equal portions. Each portion in turn was left out as a test sample, while the remaining ones were treated as training sets. During cross validation, some nodes are separated into the test sample and the corresponding links cannot be predicted by LPBIN; therefore, those links were not considered in the process of evaluation. The ROC curves for LPBNI, RWR, and ProCF are shown in Figure S1, supporting the superior performance of LPBNI as well. Taken together, these analyses demonstrate the power of LPBNI in the prediction of lncRNA–protein interactions.

### Comparison with the existing methods in predicting lncRNA–protein interactions

**Figure 2:**
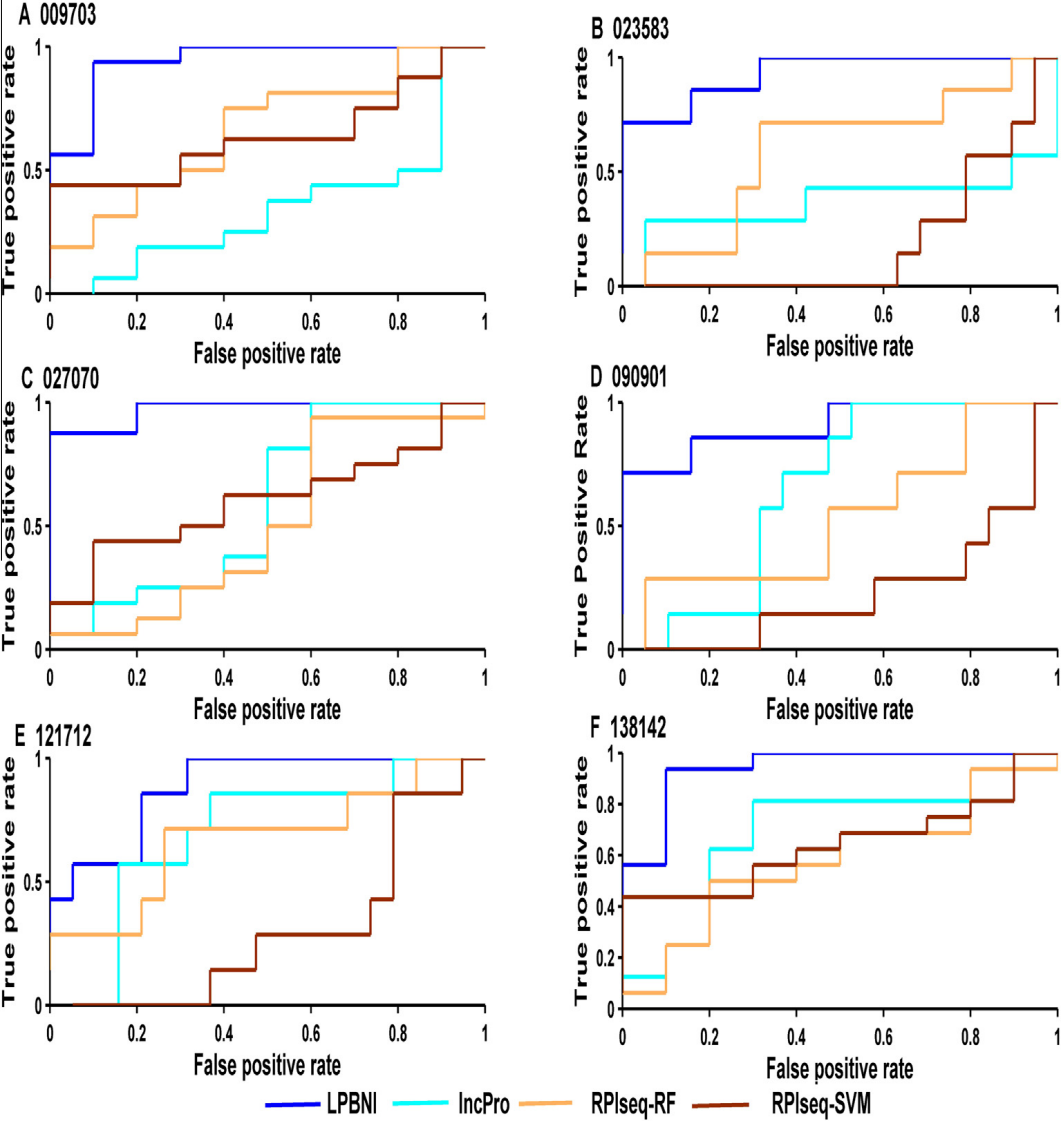
**Performance comparison in terms of ROC curves for six lncRNAs tested** Shown in the plot is the ROC curves for 6 lncRNAs (**A**–**F**) using LPBNI (blue), lncPro (cyan), RPISeq-RF (yellow), and RPISeq-SVM (brown), respectively. For each lncRNA, LOOCV is performed and the corresponding lncRNA–protein interactions are used as gold standard dataset. The lncRNA IDs have been abbreviated without the “NONHSAT” prefix, *e.g.*, “009703” represents the lncRNA “NONHSAT009703”. ROC, receiver operating characteristic.

In order to further evaluate the performance of the proposed method in predicting lncRNA–protein interactions, we compared LPBNI with lncPro [29] and RPISeq [26]. RF or SVM can be used in RPISeq [26] for the prediction of lncRNA–protein interactions, which correspond to RPISeq-RF and RPI-SVM here. We performed the evaluation of these methods using six lncRNAs, including NONHSAT009703, NONHSAT023583, NONHSAT027070, NONHSAT090901, NONHSAT121712, and NONHSAT138142, which we randomly selected from the lncRNAs set and present 69 lncRNA–protein interactions. The ROC curves for predicting lncRNA–protein interactions are shown in Figure 2. For each lncRNA, the corresponding ROC curve of LPBNI is significantly higher compared with the results obtained by lncPro, RPISeq-RF, and RPISeq-SVM. As presented in Table 3, the average AUC of LPBNI is 0.375, 0.339, and 0.497 higher than lncPro, RPISeq-RF, and RPISeq-SVM, respectively. The comparisons among LPBNI, lncPro, and RPISeq, in terms of sensitivity and specificity, are listed in Table S1. Taking NONHSAT138142 (RP6-24A23.7) as an example, the AUC value of LPBNI was shown to be 0.944, which is 0.263, 0.363, and 0.300 higher compared with lncPro, RPISeq-RF, and RPISeq-SVM, respectively. For specificity of 95.0%, the sensitivity of LPBNI was 0.563, which is 0.438, 0.500, and 0.125 higher compared with lncPro, RPISeq-RF, and RPISeq-SVM, respectively.

**Figure 3:**
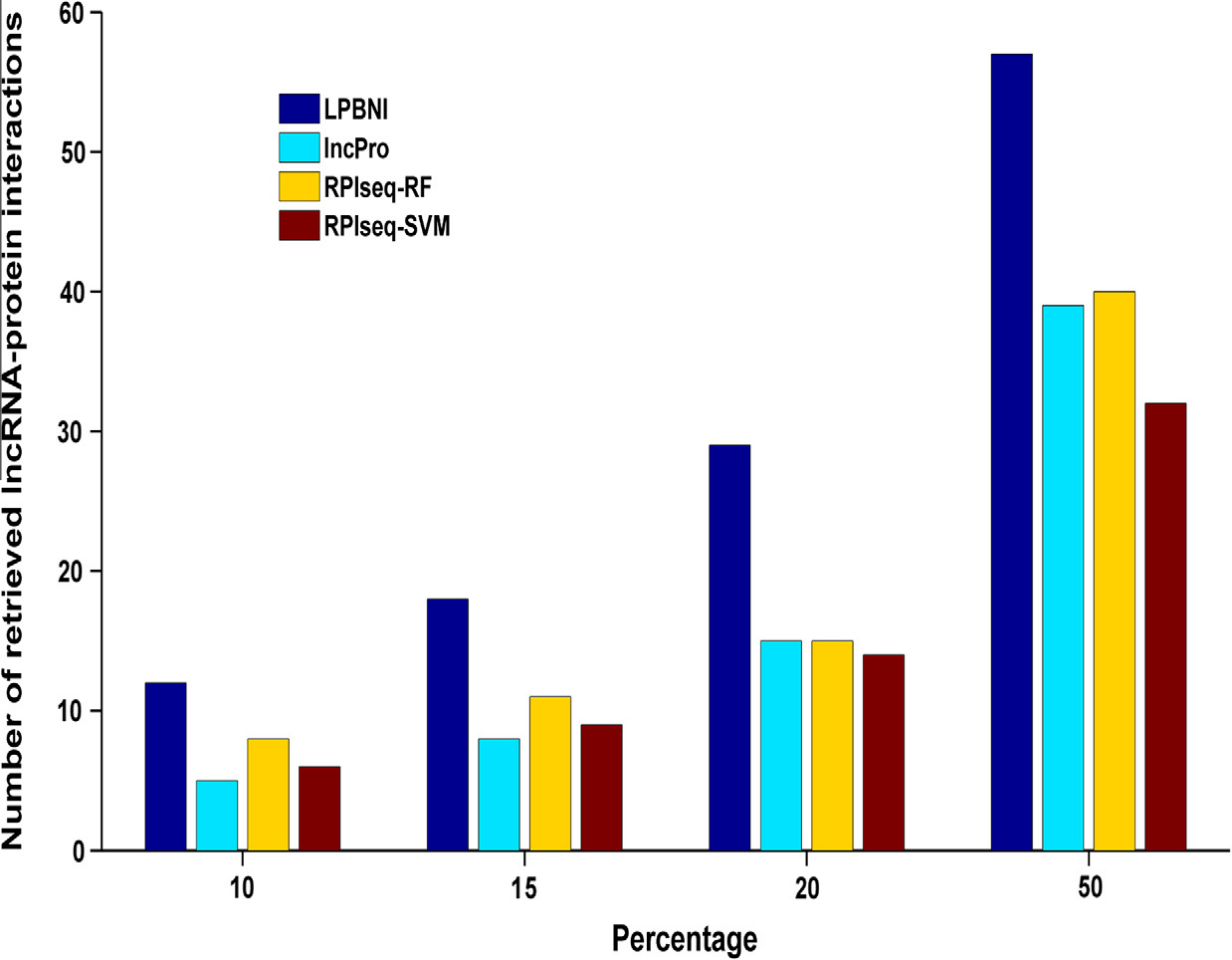
**Number of interactions that are correctly recovered from 4796 true interactions in different percentiles** 4796 known lncRNA–protein interactions are taken as gold standard dataset. 10%, 15%, 20%, and 50% of all the prediction results are taken as different percentiles. The number of lncRNA–protein interactions that are correctly retrieved at different percentiles indicated the rank distribution of the lncRNA–related proteins, the higher the number in each percentile, the better the performance of the method.

As aforementioned, there are 69 lncRNA–protein interaction pairs for these six lncRNAs. We next examined the numbers of lncRNA–protein interactions that were correctly recovered with respect to different percentiles [32] of all the prediction results. As shown in Figure 3, LPBNI had the best performance for every percentile tested. Top-ranked results are of great importance, due to the low occurrence of false positive results. When looking at the top 10% of the results, 12 of 69 lncRNA–protein interactions were correctly retrieved by LPBNI, whereas only five, eight, and six interactions were correctly retrieved by lncPro, RPISeq-RF, and RPISeq-SVM, respectively. As for top 50%, LPBNI correctly recovers 57 lncRNA–protein interactions, which represent 18, 17, and 25 interactions more than lncPro, RPISeq-RF, and RPISeq-SVM, respectively. These comparisons indicate that LPBNI outperforms lncPro and RPISeq in the prediction of lncRNA–protein interactions.

### Prediction of novel lncRNA–protein interactions

Following the validation of the superior performance of LPBNI using LOOCV, we applied LPBNI onto the 4796 known lncRNA–protein interactions downloaded from NPInter [34], which includes 1113 lncRNAs and 26 proteins to predict novel lncRNA–protein interactions. For each lncRNA, all the collected proteins were ranked according to the scores calculated by LPBNI, and the top five proteins are considered potential lncRNA–interacting proteins. We present here the results for four lncRNAs, which include NONHSAT037119 (RP11-349A22.5), NONHSAT010657 (HNRNPU-AS1), NONHSAT016118 (RP11-18I14.10), and NONHSAT027801 (RP11-350F4.2). Top five proteins and the corresponding scores for these lncRNAs are presented in Table 4. We searched other databases such as starBase [35] and lncRNome [36], and found that some top ranked proteins that are predicted to interact with these lncRNAs are supported by starBase [35], which is designed to decipher miRNA-target interactions and protein-RNA interactions. As shown in Table 4 and 9606.ENSP00000254108 (RNA-binding protein FUS), 9606.ENSP00000401371 (Nucleolysin TIA-1 isoform p40), and 9606.ENSP00000349428 (Polypyrimidine tract-binding protein 1) are predicted to interact with RP11-349A22.5. 9606. ENSP00000290341 (Insulin-like growth factor 2 mRNA-binding protein 1), and 9606.ENSP00000258962 (Serine/arginine-rich splicing factor 1), and 9606.ENSP00000350028 (Putative helicase MOV-10) are predicted to interact with HNRNPU-AS1. FUS is predicted to interact with RP11-18I14.10 and RP11-350F4.2. These predictions were all confirmed by starBase [35].

Furthermore, we extracted the predictions confirmed by starBase and compared their ranks predicted by LPBNI, lncPro, RPISeq-RF, and RPI-SVM (Table 5). The results showed that for these lncRNAs, there exist large differences in the ranks of most of the candidate proteins predicted by LPBNI, lncPro, RPISeq-RF, and RPI-SVM. Despite these great variations, candidate proteins are consistently ranked higher by LPBNI relative to the other three methods. For instance, for lncRNA RP11-350F4.2, FUS is ranked first by LPBNI, but it is ranked 12th, 15th, and 24th by lncPro, RPISeq-RF, and RPI-SVM, respectively. The results above show that LPBNI can identify potential lncRNA–interacting proteins as top candidates, implying that the use of LPBNI is a very effective way to predict novel lncRNA–protein interactions.

## Discussion

In this study, we proposed and tested a novel computational method, LPBNI, for the prediction of potential lncRNA–protein interactions. We constructed an lncRNA–protein bipartite network, using the information about lncRNA–protein interactions, lncRNA and proteins are connected if they were known to interact with each other. Following this, two-step propagation was carried out in the bipartite network to score and rank candidate proteins for each lncRNA. The proposed method has some important features. Firstly, LPBNI uses only the network constructed based on the known lncRNA–protein interactions to perform this prediction. Secondly, with an increasing degree of a node, less information is assigned to its direct neighbors. Finally, the propagation matrix is not symmetrical. The results of comparisons between LPBNI and other network-based methods show that LPBNI has higher AUC, compared to RWR and ProCF. In order to further evaluate the performance of the proposed method, we compare LPBNI with the existing methods for lncRNA–protein pair prediction and obtain consistently higher ROC curves using LPBNI in relative to lncPro, RPISeq-RF, and RPISeq-SVM for the six lncRNAs tested. All the comparisons show that our method can effectively predict interactions between lncRNAs and proteins, largely by taking advantage of lncRNA–protein interaction network. The case study shows further that LPBNI is powerful not only for the recovery of known lncRNA–protein interactions, but also for the prediction of potential candidate proteins. This suggests that LPBNI may be a useful tool for predicting candidate lncRNA–interacting proteins that could be subjected to further experimental investigation for potential functional studies.

Despite the efficiency of LPBNI in the prediction of the candidate proteins for interacting with lncRNAs, some limitations exist. Firstly, LPBNI can only be implemented for a bipartite network, in which each node has at least two links. Since LPBNI only uses the prior information about the known lncRNA–protein interactions, LPBNI cannot predict candidate proteins if there is no information about the lncRNAs in the training set. This limitation may be addressed by extending the bipartite network to a bipartite network model based on lncRNA/protein functional domains or by adding the expression profile of lncRNAs [38]. Secondly, some proteins interact with a lot of lncRNAs, which may tend to get more information during the procedure of information propagation, and in consequence have higher scores in the prediction. Finally, the shortage of known lncRNA–protein interactions limits the further analysis of lncRNA mechanisms in a larger network, which may be addressed by a rapid increase in lncRNA datasets.

## Conclusion

The prediction of lncRNA–protein interactions is extremely important for the studies of the complex function of lncRNAs. Existing methods are using the sequence information of lncRNAs and proteins, but in this study, we introduce a network-based method, LPBNI, which takes full advantage of the information about the known lncRNA–protein interactions. We performed the evaluation and case study of this method, which further demonstrate its superior performance.

## Materials and methods

### Data collection and preprocessing

7576 ncRNA–protein interactions were downloaded from the NPInter 2.0 database [34] (http://www.bioinfo.org/NPInter/) in November, 2013, with the restriction of type “NONCODE” and organism “*Homo sapiens*”. Furthermore, we extracted 2380 lncRNAs from a human lncRNA dataset downloaded from NONCODE 4.0 database [39], and converted the IDs of lncRNAs and proteins, into NONCODE 4.0 IDs and string IDs, separately. Finally, we got 4870 lncRNA–protein interactions, including 2380 lncRNAs and 106 proteins.

### The lncRNA–protein bipartite network

**Figure 4:**
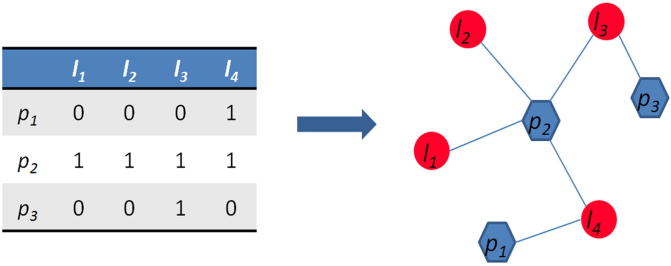
**Example of the lncRNA–protein interaction bipartite network** The left panel is the adjacent matrix *A* extracted from the known lncRNA–protein interactions, where *a_ij_* = 1 if *p_i_* interacts with *l_j_*, otherwise *a_ij_* = 0. The right panel is the bipartite network constructed based on the adjacent matrix. The red circles represent lncRNAs and blue hexagons represent proteins, while the known lncRNA–protein interactions are represented in blue lines.

The lncRNA–protein interaction network can be described as a graph *G*(*L,P,E*), in which *L* = {*l_1_*,*l_2_*,…,*l_n_*} is defined as lncRNA set, *P* = {*p_1_*,*p_2_*,…,*p_m_*} is defined as the protein set, and *E* *=* {*e_i,j_|L_i_∈L,P_j_∈P*} is the edge set, where *e_i,j_* represents the edge connecting the nodes *p_i_* and *l_j_*. *A* *=* {*a_i,j_|i∈P,j∈L*} represents the adjacent matrix, where *a_ij_* = 1 if *p_i_* interacts with *l_j_*, otherwise *a_ij_* = 0. For lncRNA *l_j_*, positive samples referred to the proteins that are known to interact with *l_j_*, and the remaining proteins were considered negative samples. A simple illustration of lncRNA–protein bipartite network construction is shown in Figure 4. Finally, the network was constructed and a propagation method was applied to compute the interaction score.

### LPBNI method

**Figure 5:**
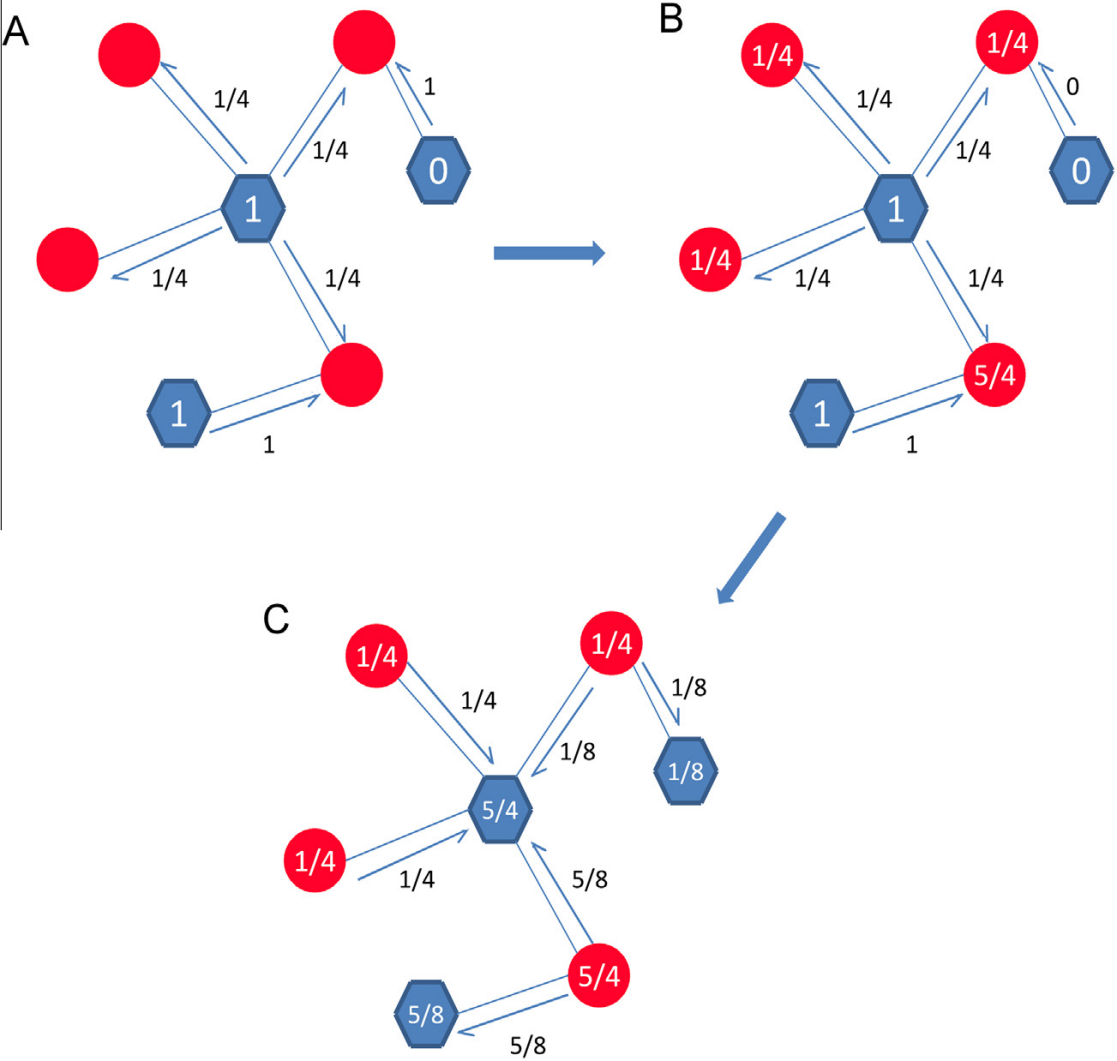
**Illustration of the LPBNI in bipartite network** **A.** The process of the initial information propagated from proteins to their direct neighbor lncRNAs. For example, the initial information of three proteins is 1, 1 and 0, respectively. **B.** The score on red circles is the information of each lncRNA received from proteins. **C.** The process of the information propagated from lncRNAs back to proteins. The score on blue hexagon in panel C is the final information of each protein after the two-step propagation. The red circles represent lncRNAs and the blue hexagons represent proteins.

The propagation process of LPBNI was derived from the recommendation algorithms proposed by Zhou et al. [40], and developed for the prediction of lncRNA–protein interactions. The proposed method makes full use of the information about the known lncRNA–protein interactions, and scores candidate proteins for each lncRNA. We classified the nodes of lncRNA–protein interaction network into two different sets, named *P* and *L* as aforementioned and only the connections between different sets were allowed. LPBNI procedure is illustrated in Figure 5. For example, if the initial information of three proteins was 1, 1, and 0, we first propagated information from proteins to the corresponding lncRNAs. Afterward, the information was allocated from lncRNAs back to proteins. Since the network is unweighted, the information in a protein is equally propagated to its direct neighbors in the lncRNA set, and *vice versa*. The propagation of information after each step is shown in Figure 5B and C, respectively. This two-step propagation can be represented as:

(1) $$\left(\begin{array}{c} 5/4\\ 5/8\\ 1/8 \end{array}\right)=\left(\begin{array}{ccc} 3/4 & 1/2 & 1/2\\ 1/8 & 1/2 & 0\\ 1/8 & 0 & 1/2 \end{array}\right)\left(\begin{array}{c} 1\\ 1\\ 0 \end{array}\right)$$

Here, 3 × 3 matrix represents the propagation matrix. Following the two-step propagation on the bipartite network, the final information of these three proteins becomes 5/4, 5/8, and 1/8, respectively.

For the lncRNA–protein interaction bipartite network *G*(*L,P,E*), *W* is defined as the propagation matrix, where *w_ik_* represents the information transferred from *p_k_* to *p_i_* node, and can be interpreted as the importance of *p_i_* for node *p_k_*. For an lncRNA *l_j_*, we define *S*_0_(*i*) = *s_ij_*, *i∈*{1,2…*m*} as the initial information of protein *P*, *s_ij_* = 1 if *p_i_* interacts with *l_j_*, otherwise *s_ij_* = 0. *S_L_*(*l_j_*), *j∈*{1,2…*n*} represents the score on *l_j_* after the first step of information propagation, which can be calculated as:

(2) $$S_{L}(l_{j})=\overset{m}{\underset{i=1}{\sum }}\frac{a_{\mathrm{ij}}S_{0}(i)}{d(p_{i})}$$

where $d(p_{i})=\sum_{j=0}^{n}a_{\mathrm{ij}}$ is the number of lncRNAs that interact with *p_i_.*

In the second step, all the information in *L* propagates back to *P*. *S_F_*(*p_i_*) is defined as the final information of protein *p_i_*, representing the interaction score of protein *p_i_* with *l_j_*. *S_F_* can be defined as

(3) $$S_{F}(i)=\overset{n}{\underset{j=1}{\sum }}\frac{a_{\mathrm{ij}}S_{L}(l_{j})}{d(l_{j})}=\overset{n}{\underset{j=1}{\sum }}\frac{a_{\mathrm{ij}}}{d(l_{j})}\overset{m}{\underset{k=1}{\sum }}\frac{a_{\mathrm{kj}}S_{0}(k)}{d(p_{k})}$$

where $d(l_{j})=\sum_{1=0}^{m}a_{\mathrm{ij}}$) is the number of proteins that interact with *l_j_*.

The final information *S_F_* can be defined in the matrix form as

(4) $$\overset{\to }{S_{F}}=W\overset{\to }{S_{0}}$$

where $\overset{\to }{S_{0}}$ is the column vector of *S*_0_, and $\overset{\to }{S_{F}}$ is the final score of query lncRNA after the two-step information propagation in lncRNA–protein interaction network. Eq. (3) can be represented as:

(5) $$S_{F}(i)=\overset{m}{\underset{k=1}{\sum }}w_{\mathrm{ik}}S_{0}(k)$$

where

(6) $$W_{\mathrm{ij}}=\frac{1}{d(p_{i})}\overset{n}{\underset{j=1}{\sum }}\frac{a_{\mathrm{ij}}a_{\mathrm{kj}}}{d(l_{j})}$$

Following the calculations, the proteins were ranked for *l_j_* by the final score *S_F_*. All of the candidate proteins are listed in a descending order, and highly-ranked proteins are considered to interact with lncRNA *l_j_*. The data and source code are freely available at https://github.com/USTC-HILAB.

### Experimental design

LOOCV was performed on the lncRNA–protein interaction network for performance evaluation of the proposed method. In this process, each lncRNA–protein pair was left out in turn as a test sample, by setting the corresponding value in the adjacent matrix *A* to 0. The performance of LPBNI was estimated by the success rate it achieves in recovering the known lncRNA–protein interactions. In order to assess the performance of LPBNI, we plotted the ROC curves, and compared the AUC values obtained using LPBNI, RWR, and ProCF. Additionally, we computed *Sp*, *Sn*, *Acc*, *Pre*, and *MCC* values. The propagation matrix *W* presented in this paper relies on the adjacent matrix *A* of the bipartite network. When LOOCV was implemented, we obtained different *W* values, due to the change of *A* values in each step of LOOCV. Therefore, *W* value was recalculated for each lncRNA–protein pair that was left out as test sample. Furthermore, during LOOCV process, no information was propagated on the nodes with less than two links, and these nodes were not considered during the performance evaluation.

## Authors’ contributions

MG participated in the downloading and preprocessing of the datasets, carried out the design and performance evaluation of LPBNI in predicting lncRNA–protein interactions. AL conceived of the project and helped with the study design. MW was involved in data analysis. MG drafted the manuscript with the help of AL and MW. All authors read and approved the final manuscript.

## Competing interests

The authors have declared no competing interests.

## Figures and Tables

**Table 1:** Performance comparison of different methods with specificities of 99.0% and 95.0% in predicting lncRNA–protein interactions

**Specificity**	**Methods**	**Sensitivity**	**Accuracy**	**Precision**	**MCC**
99.0%	LPBNI	0.288	0.873	0.852	0.449
RWR	0.062	0.835	0.556	0.282
ProCF	0.118	0.844	0.703	0.334

95.0%	LPBNI	0.532	0.880	0.681	0.534
RWR	0.156	0.817	0.384	0.480
ProCF	0.317	0.844	0.560	0.528

*Note:* LPBNI, lncRNA–protein bipartite network inference; RWR, random walk; ProCF, protein-based collaborative filtering; MCC, Matthew’s correlation coefficient.

**Table 2:** Number of interactions that are correctly recovered from 69 true interactions using different methods

**Method**	**No. of interactions recovered at each correct recovery percentile**				
**5%**	**10%**	**15%**	**20%**	**50%**
LPBNI	579	1031	1543	2566	4269
RWR	410	943	1409	2180	4092
ProCF	116	326	620	1180	2826

*Note*: Comparison was performed at different correct recovery percentiles including 5%, 10%, 15%, 20%, and 50%.

**Table 3:** AUC comparison of different methods for six lncRNAs

**lncRNA ID**	**LPBNI**	**lncPro**	**RPISeq-RF**	**RPISeq-SVM**
NONHSAT009703	0.944	0.344	0.663	0.638
NONHSAT023583	0.932	0.368	0.594	0.188
NONHSAT027070	0.975	0.594	0.506	0.606
NONHSAT090901	0.910	0.654	0.534	0.233
NONHSAT121712	0.887	0.699	0.677	0.301
NONHSAT138142	0.944	0.681	0.581	0.644

*Note:* AUC, area under the curve.

**Table 4:** Top 5 ranked candidate proteins for four selected lncRNAs

**lncRNA ID (NONCODE 4.0 ID)**	**Top 5 ranked proteins**		**LPBNI score**	**Validated**
**STRING ID**	**Name**		
NONHSAT037119 (RP11-349A22.5)	9606.ENSP00000254108	RNA-binding protein FUS	0.528	starBase
9606.ENSP00000220592	Signal recognition particle 54 kDa protein	0.278	
9606.ENSP00000240185	TAR DNA-binding protein 43	0.275	
9606.ENSP00000401371	Nucleolysin TIA-1 isoform p40	0.247	starBase
9606.ENSP00000349428	Polypyrimidine tract-binding protein 1	0.192	starBase

NONHSAT010657 (HNRNPU-AS1)	9606.ENSP00000290341	Insulin-like growth factor 2 mRNA-binding protein 1	0.801	starBase
9606.ENSP00000240185	TAR DNA-binding protein 43	0.483	
9606.ENSP00000258962	Serine/arginine-rich splicing factor 1	0.315	starBase
9606.ENSP00000350028	Putative helicase MOV-10	0.304	starBase
9606.ENSP00000338371	Trinucleotide repeat-containing gene 6B protein	0.144	

NONHSAT016118 (RP11-18I14.10)	9606.ENSP00000385269	ELAV-like protein 1	0.396	
9606.ENSP00000254108	RNA-binding protein FUS	0.175	starBase
9606.ENSP00000220592	Signal recognition particle 54 kDa protein	0.121	
9606.ENSP00000381031	RNA-binding protein EWS	0.096	
9606.ENSP00000401371	Nucleolysin TIA-1 isoform p40	0.087	

NONHSAT027801 (RP11-350F4.2)	9606.ENSP00000254108	RNA-binding protein FUS	0.440	starBase
9606.ENSP00000240185	TAR DNA-binding protein 43	0.286	
9606.ENSP00000220592	Signal recognition particle 54 kDa protein	0.276	
9606.ENSP00000381031	RNA-binding protein EWS	0.268	
9606.ENSP00000349428	Polypyrimidine tract-binding protein 1	0.187	

*Note:* The corresponding score of each interaction is calculated by LPBNI. The higher the score, the higher possibility of the protein interacts with the query lncRNA. For each lncRNA, the proteins were ranked in a descending order based on the score.

**Table 5:** Top candidate proteins predicted by LPBNI with support by starBase and their ranks predicted using different methods

**lncRNA ID (NONCODE 4.0 ID)**	**Candidate proteins**		**Rank using each method**			
	**STRING ID**	**Name**	**LPBNI**	**lncPro**	**RPISeq-RF**	**RPISeq-SVM**
NONHSAT037119(RP11-349A22.5)	9606.ENSP00000254108	RNA-binding protein FUS	1	9	2	24
9606.ENSP00000401371	Nucleolysin TIA-1 isoform p40	4	4	16	6
9606.ENSP00000349428	Polypyrimidine tract-binding protein 1	5	23	11	10

NONHSAT010657(HNRNPU-AS1)	9606.ENSP00000290341	Insulin-like growth factor 2 mRNA-binding protein 1	1	3	21	21
9606.ENSP00000258962	Serine/arginine-rich splicing factor 1	3	15	3	19
9606.ENSP00000350028	Putative helicase MOV-10	4	4	15	15

NONHSAT016118 (RP11-18I14.10)	9606.ENSP00000254108	RNA-binding protein FUS	2	15	8	26

NONHSAT027801 (RP11-350F4.2)	9606.ENSP00000254108	RNA-binding protein FUS	1	12	15	24

## Supplementary material

Supplementary Table S1 Comparison of specificity and sensitivity for six lncRNAs using different methods
