# Supplementary material for: A Bipartite Network-based Method for Prediction of Long Non-coding RNA–protein Interactions
Source: Genomics Proteomics Bioinformatics. 2016 Feb 22;14(1):62–71. doi: 10.1016/j.gpb.2016.01.004 (PMC4792848; doi:10.1016/j.gpb.2016.01.004)
Supplement: Supplementary Table S1 — Comparison of specificity and sensitivity for six lncRNAs using different methods [file mmc1.docx]

**Table S1 Comparison of specificity and sensitivity for six lncRNAs using different methods**

| **lncRNA ID** | **Sensitivity (with a specificity of 95%)** | | | | **Sensitivity (with a specificity of 90%)** | | | |
| --- | --- | --- | --- | --- | --- | --- | --- | --- |
|  | **LPBNI** | **lncPro** | **RPIseq-RF** | **RPIseq-SVM** | **LPBNI** | **lncPro** | **RPIseq-RF** | **RPIseq-SVM** |
| NONHSAT009703 | 0.563 | 0 | 0.188 | 0.438 | 0.563 | 0 | 0.188 | 0.438 |
| NONHSAT023583 | 0.714 | 0 | 0 | 0 | 0.714 | 0.286 | 0.143 | 0 |
| NONHSAT027070 | 0.875 | 0.063 | 0.063 | 0.189 | 0.875 | 0.063 | 0.063 | 0.189 |
| NONHSAT090901 | 0.714 | 0 | 0 | 0 | 0.714 | 0 | 0.286 | 0 |
| NONHSAT121712 | 0.429 | 0 | 0.286 | 0 | 0.571 | 0 | 0.286 | 0 |
| NONHSAT138142 | 0.563 | 0.125 | 0.063 | 0.438 | 0.563 | 0.125 | 0.063 | 0.438 |
